# Chemokine-Like Factor 1-Derived C-Terminal Peptides Induce the Proliferation of Dermal Microvascular Endothelial Cells in Psoriasis

**DOI:** 10.1371/journal.pone.0125073

**Published:** 2015-04-27

**Authors:** Yaqi Tan, Yixuan Wang, Li Li, Jinyu Xia, Shiguang Peng, Yanling He

**Affiliations:** Department of Dermatology, Beijing Chao-Yang Hospital, Capital Medical University, Beijing, China; University of Sassari, ITALY

## Abstract

Psoriasis is an inflammatory disease characterized by the abnormal proliferation of skin cells, including dermal microvascular endothelial cells. Recently, chemokine-like factor 1 (CKLF1) was found to participate in the local inflammation and cell proliferation. To explore its role in the pathogenesis of psoriasis, the expression of both CKLF1 and its receptor (CCR4) was determined in the psoriatic lesions. Also, the effect of the C-terminal peptides (C19 and C27) of CKLF1 on the proliferation of human umbilical vein endothelial cells was studied in vitro. By immunohistochemistry and immunofluorescence, the expression of both CKLF1 and CCR4 was determined in the psoriatic lesions. The effect of C-terminal peptides on human umbilical vein endothelial cells (HUVECs) was studied in vitro by the evaluation of cell proliferation and apoptosis. The in vivo assessment was performed accordingly through the subcutaneous injection peptides on BALB/c mice. The results showed that, by immunohistochemistry, both CKLF1 and CCR4 were increasingly expressed in psoriatic lesions as compared to normal skins. Moreover, the primary umbilical vein endothelial cells exhibited higher proliferation ratio under the C19 or C27 stimulation, which was even enhanced by the addition of psoriatic sera or TNF-α. Furthermore, the enhancement of peptide simulation was accompanied with the activation of ERK1/2-MAPKs pathway. In addition, such effect of C19 and C27 was mirrored by the hyperproliferation of cutaneous microvessels in BALB/c mice that were subcutaneously injected with the two peptides. Therefore, we concluded that CKLF1 plays a role in the pathogenesis of psoriasis by promoting the proliferation of microvascular endothelial cells that possibly correlates with ERK1/2-MAPKs activation.

## Introduction

Psoriasis is an inflammatory skin disorder characterized by the epidermis hyperproliferation, inflammatory cell accumulation, and dilation of dermal papillary blood vessels [1]. The formation of new vessels begins during early psoriatic stage and disappears with clearance of the disease. Pro-angiogenic cytokines, especially tumor necrosis factor (TNF), are up-regulated in the psoriasis development [2, 3]. Mediator-dependent factors derived from the keratinocytes or other immune cells may contribute to the proliferation of dermal microvascular endothelial cells that is pivotal in the vessel formation. Over recent decades, more supportive evidences have shown that certain chemokines and their receptors are involved in the progression of psoriatic lesion, possibly by participating in the process of leukocyte recruitment and activation at the sites of local inflammation [4–7]. Actually, these molecules also regulate endothelial cell functions, such as proliferation, migration, angiogenesis, and adhesion molecule expression, all of which contribute to the manifestations of psoriasis [8–10]. From the environment endothelial cells receive multiple information that eventually leads to such progress as above, following the signal transduction through triggering the cascade pathways and downstream network of cross-talks. Therefore, to investigate the function of these cytokines in regulating microvascular endothelial cells is helpful for not only the elucidation of pathogenesis but also the exploration of novel therapeutic targets.

Chemokine-like factor 1 (CKLF1) is a newly discovered cytokine which functions by engaging its receptor of C-C chemokine receptor 4 (CCR4) [11]. The full length of CKLF1 cDNA has been cloned from phytohaemagglutinin-stimulated U937 cells, and encodes ninety-nine amino residues [12]. Purified recombinant CKLF1 protein from a *Drosophila* expression system revealed that CKLF1 contained at least two secreted peptides located at its C-terminal, named C19 and C27 [13]. It was found that chemically synthesized C19 and C27 also exhibit functional activity via CCR4 [14] although C27 has stronger effect on CCR4-mediated chemotaxis than C19 ^13,15^. Moreover, both C19 and C27 can abrogate the effect of CKLF1 on cells by competing for CCR4 receptor [15–17].

CKLF1 has broad-spectrum biological functions in inflammation and immune-related diseases, including asthma and atopic dermatitis [18, 19]. It has been found that CKLF1 exhibits chemotactic effects on multiple leukocytes [12, 20]. Moreover, CKLF1 can stimulate the proliferation of skeletal or vascular muscle cells, and the migration of neuroblastoma cells [21–23], and the differentiation of monocytes [24]. The effect of CKLF1 on cells might be due to the regulation of pro-apoptotic or differentiation-related proteins [21, 24, 25]. These findings strongly indicated that CKLF1 participate in the cell cycle progression and functions actively under inflammatory environment. However, up to date, the role of CKLF1 remains unclear in the pathogenesis of psoriasis, which is featured with intense inflammatory responses. This study was designed to explore the roles of CKLF1 and derived C-terminal peptides in the pathogenesis of psoriasis.

## Methods

### Tissue samples

The lesional skins were obtained from the patients with psoriasis vulgaris (11 males and 4 females, aged 30–50 years with a median of 40 years) who had been diagnosed both clinically and pathologically but received neither pharmaceutical nor physical therapy. The site-, gender-, and age-matched normal skins were collected from the patients undergoing routine surgery but without cutaneous or inflammatory-mediated diseases. The tissue samples were formalin fixed and then embedded in paraffin, whereas some fresh tissues were surgically dissected of free subcutaneous fat before freezing at -80°C [26]. Serum samples were also harvested from these patients accordingly. The study was approved by the Medical Ethics Committee of Beijing Chao-yang Hospital. Written informed consent was obtained from the patients.

### Immunohistochemistry

Hematoxylin and Eosin (H&E) staining was performed routinely [27]. For immunohistochemical staining, paraffin sections were processed by following a standard approach [28]. Briefly, after dewaxing, sections were antigen-retrieved by a high pressure method. Then, sections were incubated with mouse anti-human CKLF1 IgG (courtesy of Prof. Wenling Han, Peking University, China) or goat anti-human CCR4 IgG (Abcam, Cambridge, UK). The horseradish peroxidase-conjugated secondary antibodies (Abcam) were applied to the sections accordingly. Finally, 3,3'-diaminobenzidine-chromogen substrate was used for the color development. The isotype controls of CKLF1 or CCR4-specific IgGs were also used in immunohistochemistry.

### Immunofluorescence

Fluorescent detection was performed on frozen sections. After heat-mediated antigen retrieval as above, sections were blocked with 10% rabbit serum in phosphate buffered saline (PBS). Goat anti-human CCR4 and mouse anti-human CD31 IgGs (Abcam) were used as the primary antibodies. The detection antibodies were fluorescein isothiocyanate (FITC)-labeled rabbit anti-goat and tetraethyl rhodamine isothiocyanate-labeled rabbit anti-mouse IgGs (Southern Biotech, Birmingham, AL, USA). Nuclei were counter stained with Hoechst 33342. Sections were then imaged using the type BM-LB2 fluorescence microscope (Leica, Bensheim, Germany).

### Human umbilical vein endothelial cells (HUVECs)

Primary HUVECs were cultured by consulting the method of Jaffe et al [29]. Briefly, endothelial cells isolated from freshly umbilical cords were cultured in Medium 200, which was supplemented with 2% low serum growth supplement and 10% fetal bovine serum (Cascade Biologics, Mansfield, UK). Cultured cells were identified as endothelial cells based on their morphology and the expression of VIII factor by immunofluorescence [30]. HUVECs were used for next experiments at passages 2 to 5.

For the 24-h stimulation of HUVECs, culture media (with 2% FBS) were additionally supplemented with 2% human sera, TNF-α (0–20 ng/ml; Abcam), C27 (ALIYRKLLFNPSGPYQKKPVHEKKEVL) or C19 (FNPSGPYQKKPVHEKKEV L) peptide (both 10 nM; Hybio Engineering, Shenzhen, China), CCR4 antagonist 227013 (2 μM; Merck, Darmstadt, Germany), MEK1/2 inhibitor PD98059 (50 μM; Cell Signaling, Danvers, MA, USA) or their combination.

### Flow cytometry

HUVECs growing in 6-well plates were treated as above with human sera or TNF-α. Then, cells were harvested and fixed in 4% paraformaldehyde solution for 20 min at 4°C. For intracellular staining, cells were permeabilized in 0.2% Triton X-100/5% bovine serum albumin-PBS for 20 min at room temperature and then washed in PBS. Following routine blocking, HUVECs were incubated with PerCP-Cy5.5 labeled mouse anti-human CCR4 or isotype control (BD Biosciences, San Jose, CA, USA). A FACSCalibur flow cytometer with CellQuest software (Becton Dickinson, Franklin Lakes, NJ, USA) was used for the data acquisition and analysis.

### CCK-8 assay

HUVECs were planted at a density of 5×10^3^ cells/well in a 96-well plate, and then stimulated as above. 12 h, 24 h, and 48 h later, cell viability was analyzed with a Cell Counting Kit (CCK-8) (Dojindo, Kumamoto, Japan) according to the instructions. 10ul CCK-8 solution was added to each well and cells were incubated at 37°C for 4h, followed by the absorbance measurement at 450 nm using an Infinite M200 type microplate reader (Tecan, Maennedorf, Switzerland).

### EdU assay

Cell proliferation was evaluated by EdU incorporation assay. Briefly, cells cultured in 24-well plates were stimulated as above. Cells were then exposed to 50 mM EdU (Ribobio, Guangzhou, China) for an additional 4 h at 37°C. After that, the cells were fixed in 4% paraformaldehyde and washed with glycine solution (2 mg/ml) for 5 min. For the permeabilization, cells were treated with 0.5% Triton X-100 for 10 min at room temperature. 100 μl of Apollo reaction cocktail was added to each well for 30 min, and the nuclei were stained with Hoechst 33342. Cells were visualized under a DMI3000B fluorescent microscope (Leica). Cell proliferation ratios were calculated using the formula of (EdU-positive cells/Hoechst-stained cells) × 100%.

### Quantitative real-time polymerase chain reaction (qRT-PCR)

qRT-PCR was performed as described previously [26]. Briefly, total RNA was extracted from fresh tissues or cell cultures by PureLink RNA kit (Invitrogen, Grand Island, NY, USA). Reverse transcription was performed with a cDNA kit (Applied Biosystems, Carlsbad, CA, USA). Finally, PCR was carried out using the 7900HT Fast PCR System (Applied Biosystems). The fluorescent dye was SYBR Green Master Mixes (Invitrogen). The primers were designed using Premier 3.0 software (PREMIER Biosoft Co., Palo Alto, CA, USA), and synthesized by Sangon Company (Shanghai, China). Their sequences were as below: 5ʹ-CCATCCAGGCCACAGA AACT-3ʹ (forward) and 5ʹ- TGCGTGTAAGATGAGCTGGG-3ʹ (reverse) for CCR4; 5ʹ-AGGGGTGTTTGCACTTGTGA-3ʹ (forward) and 5ʹ-GACCGCTGGGATTGAA CAGA-3ʹ (reverse) for CKLF1; 5ʹ-GGTGTGAACCATGAGAAGTATGA-3ʹ (forward) and 5ʹ-GAGTCCTTCCACGATACCAAAG-3ʹ (reverse) for GAPDH. The amount of PCR products was normalized with GAPDH to determine the relative expression levels for each mRNA. The expression level of the objective gene was calculated according to the formulas [31]: Target gene = 2^–ΔΔCt^ × control, where ΔΔCt = (Ct_target gene_—Ct _reference gene_) _treat group_—(Ct_target gene_—Ct_reference gene_) _control group_.

### Western blotting

The status of ERK1/2 activation was examined by detecting tyrosine phosphorylation [32]. Cells in 6-well plates were stimulated for 24h as above. Phosphatase and other kinase inhibitors (Thermo Scientific, Waltham, MA, USA) were added during protein extraction. Cell lysates were routinely denatured, and then separated by SDS-PAGE. The samples transferred to PVDF membrane (Thermo Scientific) were blocked with 5% fat-free milk in Tris-buffered saline, followed by overnight incubation at 4°C with rabbit anti-human phospho-ERK1/2 (p-ERK1/2), total ERK1/2 or β-actin IgG (Cell Signaling). Horseradish peroxidase-conjugated mouse anti-rabbit IgG was used as secondary antibody. Reactive proteins were visualized by ECL kit (Merck). Densitometric analysis of the bands was performed using the Odyssey imaging system (Li-Cor Co., Lincoln, NE, USA).

### Animal experiments

Eight-week-old BALB/c mice were purchased from the Animal Department of Capital Medical University and were housed in a specific pathogen free facility. The housing conditions were controlled, with a temperature of 20–25°C and a 12:12 hours light: dark cycle. Mice were randomly divided into three groups that received single subcutaneous injection of normal saline (50 μl), which was added with TNF-α (4 μg) alone as control or C19 (or C27) peptide (4 μg). Skin biopsies were obtained five day later from the injection areas, and processed for paraffin sections, which were then stained with H&E and also immunohistochemically for CD31 (rabbit anti-mouse CD31 antibody, Abcam). The microvessel densities were counted by Image-Pro Plus 6.0 software (Media Cybernetics, Rockville, MD, USA), as described previously [33]. The protocols were approved by the Ethical Committee for Animal Studies at Capital Medical University.

### Statistical analyses

Statistical analyses were performed using SPSS software, version 17.0 (SPSS Inc. Chicago, IL, USA). Differences between more than two groups were calculated using one-way ANOVA. A two-tailed t-test was used when two groups were compared for statistical differences. Spearman’s rank correlation coefficients were used for analyzing dose-dependent relationship. Data were expressed as mean ± standard error of the mean. *P* < 0.05 was considered statistically significant.

## Results

### Both CKLF1 and CCR4 increase in psoriatic lesion

**Fig 1:**
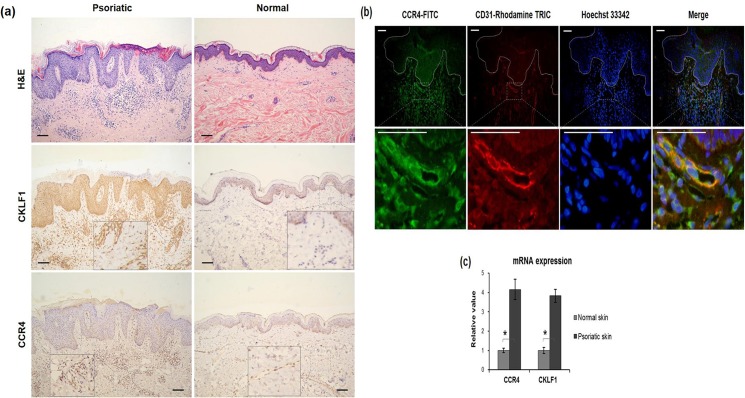
Both CKLF1 and CCR4 proteins were strongly expressed in psoriatic lesions. Paraffin sections of skin tissues were stained by H&E or immunohistochemistry for CKLF1 or CCR4. (a)As compared to normal controls, psoriatic skins exhibited stronger staining of CKLF1 in both epidermis and microvessels in dermis. Meanwhile, the dermal microvessels showed stronger CCR4 expression in psoriatic lesion than that in normal skins although no difference was found in epidermis between them. Scale bar = 100 μm. (b). Frozen sections were also analyzed for CCR4 and CD31 expression. By immunofluorescence, top panel showed the expression of CCR4 (green), CD31 (red), and nuclei (blue). Dashed white lines indicated the borders between epidermis and dermis. Bottom panel represented the magnified microvessels in dermis that expressed intense CCR4 and CD31. Scale bar = 50 μm. (c) By qRT-PCR, the mRNA levels of CKLF1 and CCR4 was found elevated in psoriatic lesion compared to the normal skins. Number of tissue samples were 15 (psoriatic) and 5 (normal), respectively. CCR4, C-C chemokine receptor 4; CKLF1, chemokine-like factor 1; H&E, hematoxylin and eosin; qRT-PCR, quantitative real-time polymerase chain reaction. * *p* < 0.05.

By immunohistochemistry, psoriatic tissues were found to express stronger CKLF1 than normal skins (Fig 1A). In the former, diffuse expression of CKLF1 was observed not only throughout the epidermis but also in the dermal microvessels that were composed of endothelial cells and infiltrated with lymphocytes. However, negative or slight staining of CKLF1 was seen in normal tissues. Furthermore, moderate positivity of CCR4 was found in epidermis from both lesional and normal skins while positively stained dermal microvessels were only shown in the leional samples (Fig 1A). The strong expression of CCR4 in psoriatic skins were also reflected by immunofluorescence that showed intense CCR4 staining in dermal microvessels (Fig 1B). Additionally, the mRNA measurement of fresh tissues confirmed significant increase in both CKLF1 and CCR4 in psoriatic skins as compared to the controls (Fig 1C).

### Psoriatic sera and TNF-α increase the CCR4 expression of HUVECs

**Fig 2:**
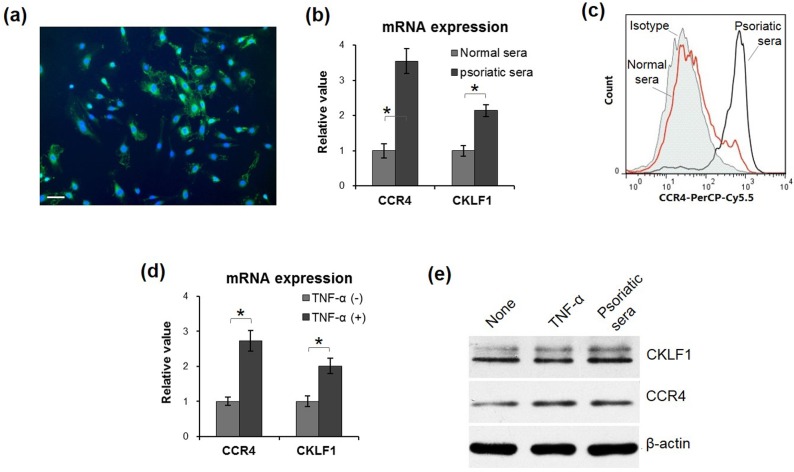
Psoriatic inflammation enhanced CCR4 and CKLF1 expression by HUVECs. (a) Primary HUVECs exhibited strong factor VIII staining (green) by immunofluorescence. Nuclei were stained by 4',6-diamidino-2-phenylindole (blue). (b) qRT-PCR confirmed elevation of mRNA levels of both CCR4 and CKLF1 upon the addition of psoriatic sera. (c) By flow cytometry, higher CCR4 expression was showed in psoriatic sera group, consistent to the qRT-PCR analysis. (d) Similarly, TNF-α displayed enhancement effect on mRNA expression of CCR4 and CKLF1 by HUVECs, which was consistent with the protein expression of CKLF1 and CCR4 (e). Data were obtained from three independent experiments. CCR4, C-C chemokine receptor 4; CKLF1, chemokine-like factor 1; HUVEC, human umbilical vein endothelial cell; qRT-PCR, quantitative real-time polymerase chain reaction; TNF-α, tumor necrosis factor-α. Scale bar = 25 μm. * *p* < 0.05.

Two days after isolation, the cells from umbilical vein exhibited morphological features of endothelial cells, including whirlpools or concentric circles of cell clones. By immunofluorescence, these cells showed positive expression of factor VIII (Fig 2A). Primary HUVECs were stimulated by normal or psoriatic sera, the latter of which had higher mRNA levels of both CCR4 and CKLF1 than the former group (Fig 2B). Consistently, flow cytometry confirmed elevated CCR4 expression by HUVECs stimulated by psoriatic sera (Fig 2C). Moreover, the TNF-α treatment exhibited enhancement effect on mRNA expression of CCR4 and CKLF1 by HUVECs (Fig 2D), which was similar to psoriatic sera. Furthermore, by western blotting, both TNF-α and CKLF1 enhanced the protein expression of CCR4 and CKLF1 (Fig 2E).

### CKLF1-derived peptides promote HUVEC viability and proliferation

**Fig 3:**
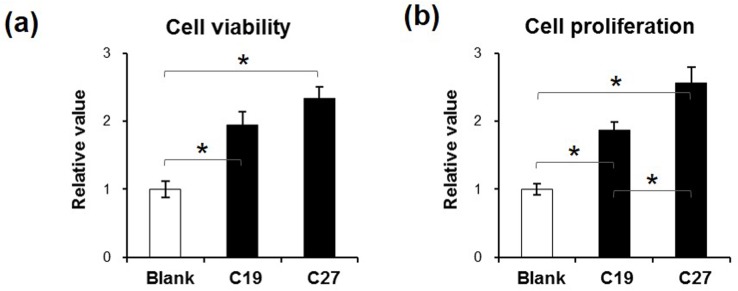
CKLF1-derived peptides promoted the viability and proliferation of HUVECs. Tumor necrosis factor-α (10 ng/ml) was added to the media, in which HUVECs were cultured for 24 h. (a) The 24-h stimulation of C19 and C27 (both 10 nM) increased the viability of HUVECs. (b) Similarly, the two peptides showed enhancement effect on cell proliferation. Compared to C19, C27 had higher potential of affection on cells. Data were obtained from three independent experiments. CKLF1, chemokine-like factor 1; HUVEC, human umbilical vein endothelial cell. *p < 0.05.

HUVECs were cultured in media supplemented with TNF-α alone or plus CKLF1-derived peptides. The results showed that cells had increased viability under peptide stimulation as compared to the control (Fig 3A and 3B). Similarly, either C19 or C27 peptide exhibited enhancement effect on HUVECs as C27 showed even greater effect on promoting proliferation (Fig 3C and 3D).

### C19 and C27 function through binding to CCR4 receptor and activating ERK1/2 pathway

**Fig 4:**
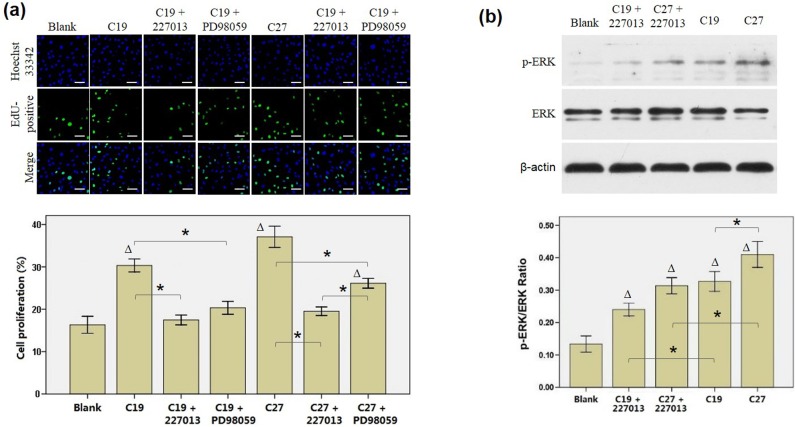
Both CCR4 antagonist (227013) and MEK1/2 inhibitor (PD98059) attenuate the effect induced by C19 and C27. HUVCEs were cultured for 24 h in media that was added with tumor necrosis factor-α (10 ng/ml) alone or plus C19 (10 nM), C27 (10 nM), 227013 (2 μM), or PD98059 (50 μM). (a) EdU assay revealed that both 227013 and PD98059 inhibited the enhancement effect of C19 and C27 on the proliferation of HUVECs. In top panel, all nuclei were stained by Hoechst (blue) while proliferating nuclei were stained by EdU (green). (b) Phosphorylated ERK (p-ERK) and total ERK were detected by Western blot from the lysates of HUVECs, showing that 227013 decreased p-ERK expression that was activated by C19 and C27 accordingly. Data were obtained from three independent experiments. HUVEC, human umbilical vein endothelial cell. Scale bar = 10 μm. * *p* < 0.05. ∆ *p* < 0.05, compared to blank group.

The peptide stimulation was also performed as culture media was added with CCR4 antagonist 227013. By EdU assay, we found that cell proliferation decreased significantly as compared to that without antagonist 227013 but similar to the blank control (Fig 4A). Moreover, the effect of MEK1/2 inhibitor PD98059 was determined, showing that the C19- or C27-induced elevation of cell proliferation was largely abrogated by PD98059 (Fig 4A). Furthermore, Western blot analysis demonstrated that both C19 and C27 increased ERK phophorylation as compared to the controls (without peptide stimulation) accordingly, while 227013 partially inhibited such effect of peptides (Fig 4B). The 227013 and PD98059 inhibition of cell proliferation proved that C19 and C27 function through binding to CCR4 receptor and activating ERK1/2 pathway, at least partially.

### Both C19 and C27 induce epidermis hyperproliferation in BALB/c mice

**Fig 5:**
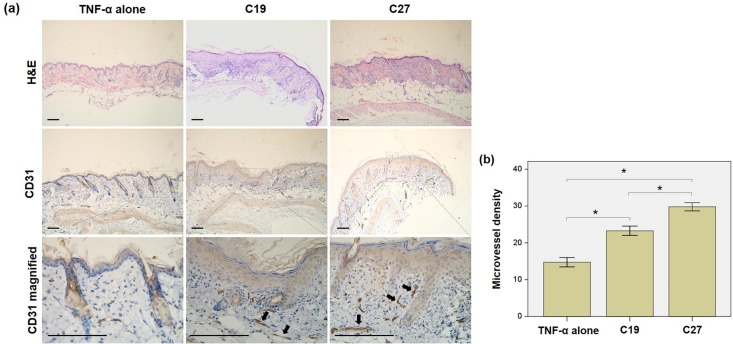
(a) Both C19 and C27 induced epidermis proliferation in BALB/c mice. Mice were subcutaneously injected with C19 or C27 peptide. The paraffin sections were prepared from the skin tissues of injection sites. a By H&E staining, the peptide groups had significantly proliferated epidermis as compared to the control (TNF-α alone). Immunohistochemistry revealed stronger CD31 staining in dermal microvessels in the peptide groups as compared to the control. (b). The microvessel densities were quantitated in CD31 stained sections, showing significant increase in the peptide-injected mice especially in C27 group. Number of mice was five in each group. H&E, hematoxylin and eosin; TNF-α, tumor necrosis factor-α. Scale bar = 100 μm.

Normal BALB/c mice were subcutaneously injected with C19 or C27 peptide. Compared to the controls (TNF-α alone), the peptide-injected skins exhibited increased proliferation of epidermis that was demonstrated by H&E staining. Additionally, immunohistochemical staining showed stronger CD31 expression in epidermis from C19- or C27-injected mice than the samples from control mice. The quantitative analysis showed higher microvessel densities (endothelial cell proliferation) in the C19 or C27 peptide injected skins when compared with the controls. See Fig 5.

## Discussion

In the present study, the results showed that both CKLF1 and CCR4 were highly expressed in the skin lesion from the patients with psoriasis. Moreover, primary HUVECs synthesized more CCR4 and CKLF1 as either psoriatic sera or TNF-α was added to culture media. In addition, CKLF1-derived peptides, C19 and C27, increased the viability and proliferation of HUVECs, which might be related to the activation of ERK1/2 pathway. Finally, the enhancement of endothelial cell proliferation was mirrored by the hyperproliferation of epidermis and dermal microvessels in BALB/c mice that were subcutaneously injected with C19 or C27 peptide.

Previously, it was found that CKLF1 may be synthesized by cells under inflammatory condition through autocrine action [34]. Our results were consistent to this finding, showing that HUVECs secrete more CKLF1 as stimulated by psoriatic sera or TNF-α. Moreover, CKLF1-derived peptides enhance the proliferation of HUVECs. Additionally, the CKLF1 peptides function through the identical receptor of CCR4. However, the reported studies also showed that CKLF1 is associated with neuronal apoptosis but not proliferation [35]. Also, the C19 and C27 peptides were reported to function reversely to protect against focal cerebral ischemia and allergic lung inflammation and inhibit leukocyte chemotaxis that were the results of CKLF1 activation [14–17, 25, 34], although the two peptides were suggested to induce the chemotaxis of HEK293 and Hut78 cells in other studies [13]. The difference between the cell fates induced by CKLF1 or derived peptides might be related to the differential origins of cells. Actually, the same chemokines may induce diversity of biological effect on different cells, which could also be the results of differences in local inflammatory environment [36, 37]. The slight discrepancy between C19 and C27 in promoting cell proliferation and activating ERK1/2 pathway might be the results of different binding affinities to CCR4 receptor or even different receptor profiles between them, which require experimental evidences in further studies.

Among the characteristics of psoriasis, the hyperproliferation of vascular endothelial cells in dermis is instrumental, and answers for the angiogenesis and formation of dilated, elongated, and tortuous capillary loops that are commonly seen in skin lesion [29]. HUVECs are widely used as a source of endothelial cells and are extrapolated to dermal microvascular endothelial cells in psoriatic dermis [38, 39]. In this study, the endothelial cells isolated from freshly umbilical cords displayed the features of HUVECs, including typical morphology and positive expression of VIII factor. Therefore, the changes of these cells reflected the status of dermal microvascular endothelial cells in psoriasis. Cytokines exhibit a profound impact on angiogenesis by influencing endothelial cell proliferation and migration. The psoriatic sera contain multiple cytokines that are correlated to the pathogenesis of psoriatic lesion, among which TNF-α is pivotal in the inflammatory network [40]. The TNF-α treatment of human keratinocytes in vitro can induce inflammatory responses in psoriasis [41]. Our results showed that HUVECs express more CKLF1 and CCR4 as stimulated by psoriatic sera or TNF-α, indicating that psoriatic inflammation contributes to the enhancement of the two components expressed by microvascular endothelial cells. In the viability and proliferation assay of cells that were treated with CKLF1-derived peptides, only TNF-α but not psoriatic sera was applied to culture media because the former provided psoriasis-like inflammation of relatively higher simplicity. Similarly, TNF-α was also used in the subcutaneous injection of C19 and C27 peptides to maintain an inflammatory condition resembling skin psoriasis. Nevertheless, both in vitro and in vivo experiments in the present study offered supportive evidence that CKLF1-derived peptides contribute to the proliferation of microvascular endothelial cells in psoriasis.

MAPK/ERK pathway is a chain of proteins in cells that communicates the signals from the receptors on cell surface to the DNA in cell nuclei. Previously, it was proven that MAPK/ERK signaling is important in the pathophysiological function of skin cells in psoriasis [42, 43]. Our results showed that MEK1/2 inhibitor PD98059 could attenuate the proliferation effect induced by C19 and C27. This finding elucidated the mechanism how CKLF1-derived peptides modulate microvascular endothelial cells through binding to the receptor of CCR4. The activation of MAPK/ERK pathway might further induce the production of pro-angiogenic factors. Therefore, to target MAPK/ERK components may lead to discovering novel therapeutic approaches in future. Besides, both C19 and C27 share somehow identical peptide sequences of CKLF1 protein. The design of therapeutic molecules that have modified structure of amino acid sequences is another aspect in the utilization of CKLF1-derived peptides.

In conclusion, our results demonstrated that both CKLF1-derived peptides of C19 and C27 promote the proliferation of dermal microvascular endothelial cells under psoriatic inflammation. The C19 and C27 peptides function through binding to the receptor of CCR4 and activating MAPK/ERK pathway. These findings contribute to the development of anti-psoriatic drugs based on the anti-angiogenesis strategies and suppression of proliferation of dermal microvascular endothelial cells.

## Supporting Information

S1 Table Demographic characteristics of the patients with psoriasis. (DOCX)
