# Supplementary material for: Chemokine-Like Factor 1-Derived C-Terminal Peptides Induce the Proliferation of Dermal Microvascular Endothelial Cells in Psoriasis
Source: PLoS One. 2015 Apr 27;10(4):e0125073. doi: 10.1371/journal.pone.0125073 (PMC4410955; doi:10.1371/journal.pone.0125073)
Supplement: S1 Table — (DOCX) [file pone.0125073.s001.docx]

**Table S1. Demographic characteristics of the patients with psoriasis**

| Patient number | Sex | Age (years) | Duration (months) | Biopsy site |
| --- | --- | --- | --- | --- |
| 1 | male | 30 | 24 | back |
| 2 | male | 42 | 48 | back |
| 3 | male | 33 | 60 | right arm |
| 4 | female | 48 | 30 | back |
| 5 | male | 41 | 12 | right thigh |
| 6 | male | 37 | 12 | back |
| 7 | female | 44 | 36 | back |
| 8 | female | 44 | 24 | left thigh |
| 9 | male | 50 | 36 | back |
| 10 | male | 38 | 10 | back |
| 11 | male | 40 | 20 | right thigh |
| 12 | male | 46 | 12 | left thigh |
| 13 | male | 35 | 24 | back |
| 14 | female | 39 | 36 | right thigh |
| 15 | male | 36 | 24 | back |
